# Supplementary material for: Disentangling metabolic functions of bacteria in the honey bee gut
Source: PLoS Biol. 2017 Dec 12;15(12):e2003467. doi: 10.1371/journal.pbio.2003467 (PMC5726620; doi:10.1371/journal.pbio.2003467)
Supplement: S8 Data — (ZIP) [file pbio.2003467.s008.zip › Z-score_plots/677 microbial product 1-Caffeoyl-4-deoxyquinic acid.pdf]

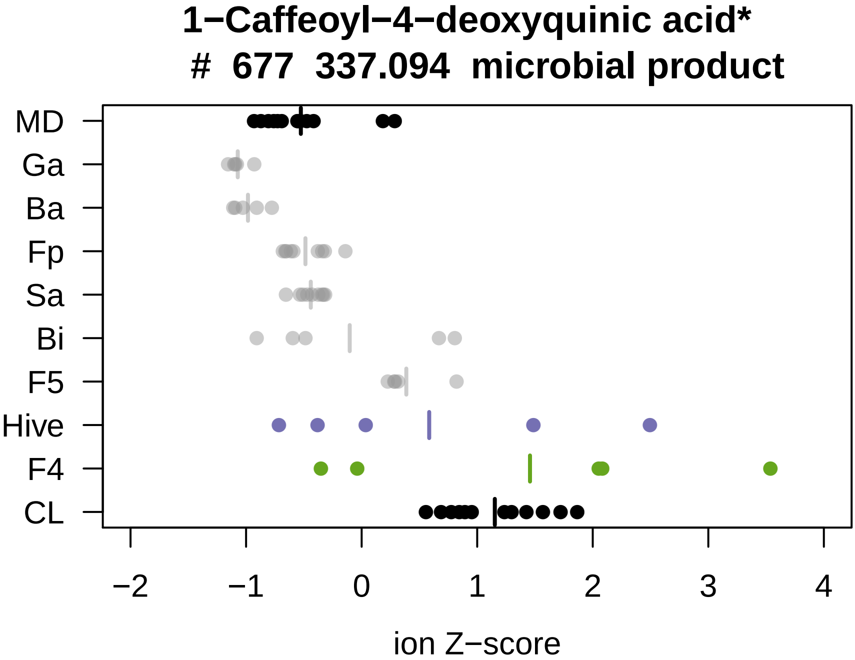
1−Caffeoyl−4−deoxyquinic acid*
677 337.094 microbial product
MD
Ga
Ba
Fp
Sa
Bi
F5
Hive
F4
CL
−2
−1
0
1
2
3
4
ion Z−score
